# Vectors for Inhaled Gene Therapy in Lung Cancer. Application for Nano Oncology and Safety of Bio Nanotechnology

**DOI:** 10.3390/ijms130910828

**Published:** 2012-08-29

**Authors:** Paul Zarogouldis, Nikos K. Karamanos, Konstantinos Porpodis, Kalliopi Domvri, Haidong Huang, Wolfgang Hohenforst-Schimdt, Eugene P. Goldberg, Konstantinos Zarogoulidis

**Affiliations:** 1 Pulmonary Department-Oncology Unit, “G. Papanikolaou” General Hospital, Aristotle University of Thessaloniki, Thessaloniki 57010, Greece; E-Mails: kporpodis@yahoo.gr (K.P.); kellybio4@hotmail.com (K.D.); zarog@med.auth.gr (K.Z.); 2 Pulmonary Department-Interventional Unit, “Ruhrland Klinik”, University of Essen, Essen 45239, Germany; 3 Laboratory of Biochemistry, University of Patras, Patras 25200, Greece; E-Mail: n.k.karamanos@upatras.gr; 4 Department of Respiratory diseases, Changhai hospital, Second Military Medical University, Shanghai 200433, China; E-Mail: hhdongbs@126.com; 5 II Medical Clinic, Hospital of Coburg, University of Wurzburg, Coburg 96450, Germany; E-Mail: w.h-s@gmx.de; 6 Biomaterials Science & Engineering, Department of Materials Science & Engineering, University of Florida, FL 32611, USA; E-Mail: egold@mse.ufl.edu

**Keywords:** lung cancer, inhaled gene therapy, non-viral vectors, nanocomplexes, nanobiotechnology

## Abstract

Novel aerosol therapeutic modalities have been investigated for lung cancer. Inhaled gene therapy has presented safety and effectiveness previously in cystic fibrosis. However, safety concerns have been raised regarding the safety of non-viral vectors for inhaled gene therapy in lung cancer, and therefore small steps have been made towards this multifunctional treatment modality. During the last decade, numerous new nanocomplexes have been created and investigated as a safe gene delivery nano-vehicle. These formulations are multifunctional; they can be used as either local therapy or carrier for an effective inhaled gene therapy for lung cancer. Herein, we present current and future perspectives of nanocomplexes for inhaled gene therapy treatment in lung cancer.

## 1. Introduction

The lung parenchyma has specific structural characteristics and properties. Thoracic malignancies, and especially lung cancer, is the leading cancer in males, with increasing incidence in women [1]. Although frequent screening of high-risk population is established by the medical community, lung cancer patients are nevertheless already at a progressed stage upon diagnosis. However, additional methods of early lung cancer detection have been evaluated and many more are on their way towards this effort [2–7]. Several treatment modalities are administered either as cytotoxic agents or immunomodulating agents for small cell lung cancer (SCLC) or non-small cell lung cancer (NSCLC) [8–12]. According to the biological pathways of lung cancer tumors, tumor specific treatment modalities are already used [13,14]. However, cancer cell mutations are being and recorded [15]. Therefore, the medical community has to investigate alternative treatments for the newly introduced mutations or resistance to current therapeutic regimens. Nevertheless, there is an alternative way of making already efficient drugs more effective and the ones that presented chemo-resistance effective once again. Local therapies for several types of cancer have demonstrated favorable results in disease control [16–20]. Lung cancer, as with any other form of cancer, is known to metastasize from the primary site, therefore a form of administration, such as aerosol, might have the additional benefit of local disease control by blocking local metastasis [21]. Aerosol administration of chemotherapeutic agents has demonstrated effectiveness, either for local endobronchial disease [22], or primary tumor mass control [19]. Although safety and efficacy has been demonstrated by many chemotherapeutic agents administered by aerosol, there are still issues to be clarified [22–28]. The main issue is whether the respiratory capacity is being altered due to the inhalational agent and whether long term scar tissue damages occur. Few studies have investigated this very important aspect in humans [18,29–32]. Two of these studies demonstrated adverse effects for doxorubicin and therefore investigation will not be further pursued for this drug formulation as an inhalation [30,31]. Another form of aerosol treatment as gene therapy has been investigated with the breakthrough of novel non-viral vectors (mainly cationic polymers). The two vector systems investigated are the viral and non-viral. Both have specific characteristics and properties. The viral vectors, although they are highly efficient in gene transfection, tend to stimulate the formation of neutralizing antibodies [33]. The non-viral vectors tend to bind more efficiently to the airway epithelium; however, they present less gene transfection and safety issues are still under investigation [34,35]. Gene therapy demonstrated safety and effectiveness in a human model in cystic fibrosis [36]. Nevertheless, due to concerns regarding the safety of the non-viral vectors, all efforts regarding aerosol gene application has been limited to *in vitro* and *in vivo* experiments in small animals [37–39]. Gene therapy as aerosol administration has been investigated mainly as tumor-suppressor gene (pathway), anti-vascular endothelial growth factor (VEGF), epidermal growth factor suppressor (EGF), K-Ras, and immune-therapy [40–43]. In the current review, we will briefly present the characteristics of the respiratory system and the aerosol properties for safe and efficient administration of gene therapy. Emphasis will be provided on the safety concerns of the non-viral vectors according to published data, and future vector delivery applications will be presented.

## 2. Search Methods

We performed an electronic article search through PubMed, Google Scholar, Medscape, and Scopus databases, using combinations of the following keywords: inhaled therapy, aerosol gene therapy in lung cancer, gene therapy vectors, gene therapy transporters, aerosol nanoparticles, inhaled therapy nanoparticles, aerosol nanocomplexes for gene therapy, gene vectors in lung cancer, and inhaled immunotherapy. All types of articles (randomized controlled trials, clinical observational cohort studies, review articles, case reports) were included. The reference lists of all included studies and review articles were checked in order to identify any further relevant citations by electronic or manual searches. Citations were reviewed without language restriction. Selected references from identified articles were searched for further consideration.

## 3. Lung Microenvironment

The lung is an organ which comes in contact directly with millions of nanoparticles through respiration. It has a vast vascular bed (100 m^2^) able to absorb drug and gas formulations and circulate them through the systemic vascular and lymphatic circulation [44,45]. To date, there is no concrete clinical data indicating which is the optimal concentration for tumor control with aerosol gene therapy (suppressor gene therapy and immunotherapy), for humans, nor whether this treatment modality will be efficient in humans. There are indications based on animal models and previously tested inhaled chemotherapy. Even in the case of inhaled chemotherapy, there are still many aspects of the tumor cells and drug formulation interaction that need to enlightened. We know the mechanisms of interaction when the drug is absorbed through the tumor vessels, but the direct interaction with the aerosol-tumor cells is yet to be defined [46]. Transporters and gene expression differ from site to site in the respiratory system and therefore the direct interaction of the aerosol-tumor cells still remains to be elicited [47,48]. To date, targeted therapy to tumors cells has been achieved by creating nanocomplexes with ligands coating various drug formulations [49,50]. There are factors that have to be evaluated when investigating a drug formulation to be administered as aerosol. The main factors to be considered are the defense mechanisms, cough, mucus, surfactant and macrophages that can prevent aerosol particles being deposited. However, macrophages play a crucial role in distributing a drug formulation to the lymphatic circulation (through phagocytosis) and therefore making the aerosol treatment more effective [51]. In specific, in lung cancer, it is crucial that apart from targeting the evident tumor mass, we need to target the micro-metastasis. In order to do that, an aerosol has to be efficiently deposited (alveoli), absorbed and circulated to the vascular circulating system and targeted to cancer cells throughout the human body. It also has to circulate through the lymphatic system and target cancer cells in lymph nodes. The aerosol also has access to the pleura through the pleura porous and further absorption to the lymphatic circulation is established through the pleura lymphatic stomata [52].

**Figure 1:**
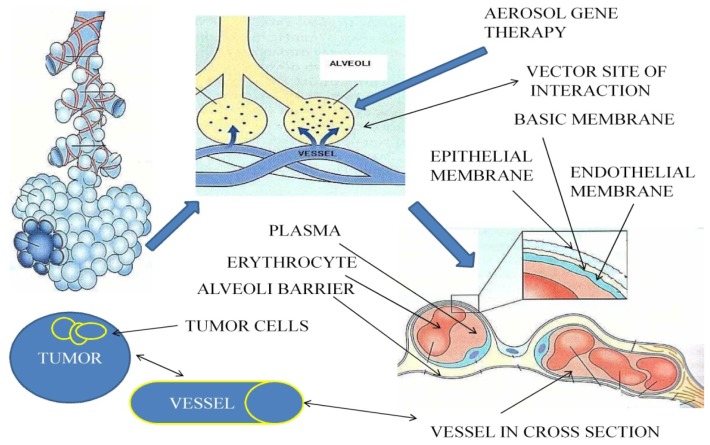
Aerosol gene therapy is administered as either liquid aerosol or dry powder. If the nanocomplex is efficiently deposited in the alveoli region it will diffuse through the alveoli membrane to the systemic circulation. It will then circulate to the lymph nodes and cancer tissue. The toxicity of the vector (if any) will be observed in the alveoli membrane. Figure by Paul Zarogoulidis.

Moreover, humidity (99.5%) is a major factor affecting almost all inhaled particles, as the particles tend to expand while moving from the upper airways to the lower [53]. Underlying lung disease (asthma, cystic fibrosis, bronchiectasis, COPD) can also affect the aerosol deposition [54]. The thick mucus production in cystic fibrosis and COPD patients blocks the proper absorption of the aerosol drug formulation by the airway epithelial [55,56]. Asthma bronchoconstriction can further disregulate the drug formulation absorption [57]. Contrariwise, in bronchiectasis, bronchial blood flow is increased, therefore increasing the distribution of the deposited aerosol [58]. Nevertheless, the aerosol product of inhaled insulin, provided indisputable data that the underlying disease is not a factor to stop or prevent its administration [59]. In the case of underlying disease, proper and intense evaluation of glucose levels have to be performed, since the drug absorption cannot be anticipated. In addition, disease control measures have to be taken, such as administration of inhaled bronchodilators, corticosteroids and *N*-acetylcysteine, to prevent exacerbations. These drugs have also been administered as preparation for inhaled chemotherapy to prevent possible adverse effects [18,19]. Finally, an aspect of the aerosol therapy regarding lung cancer that needs to be clarified is the interaction of the drug formulation with the tumor mass. Several studies conducted on the human airway model included only patients with tumor mass diameter no more than 3–5 cm. It is believed that a larger mass will have necrotic tissue and there is not going to be an interaction or absorption of the drug formulation from the cancer cells. It is assumed, but not investigated, that small tumor masses (3–5 cm in diameter) will absorb an inhaled formulation, but larger ones will not [50]. However, we should also take into consideration that the drug formulation will be deposited in normal alveoli and circulate through the vascular circulation to the vascular bed of the tumor and the rest of the human organs (Figure 1).

**Figure 2:**
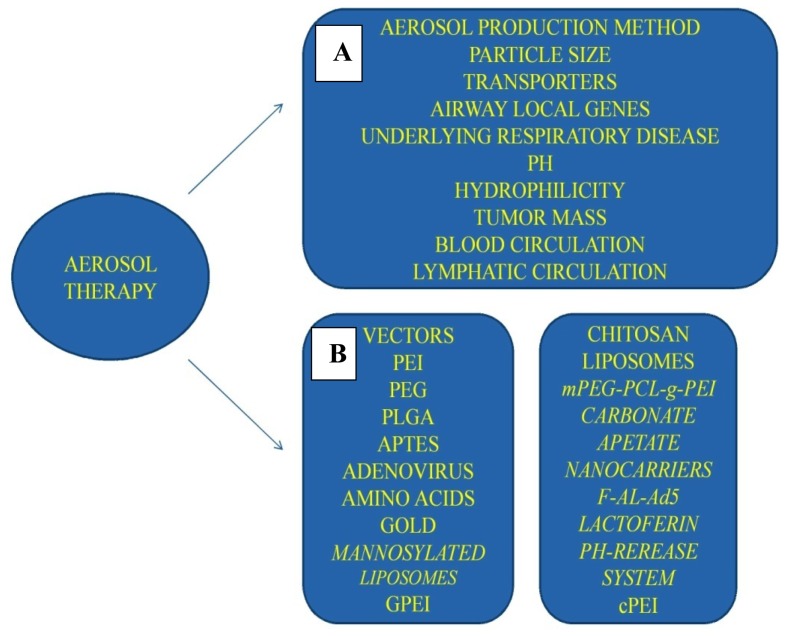
Aerosol gene therapy modality parameters; (**A**) formulation and lung microenvironment; (**B**) current and future vector systems. Figure by Paul Zarogoulidis.

It should be clarified that the reason we need local aerosol therapy for lung cancer is to deliver a smaller amount of therapeutic agent and therefore have less systemic side effects. In addition, micro-metastasis will be controlled either locally in the lung parenchyma, or in other organs through the vascular and lymphatic circulation of the chemotherapeutic agent [19]. Another aspect that should be incorporated in this section is the modification of the respiratory properties. It has been shown that the addition of 5%–7% CO_2_ through the nebulization process (aerosol gene therapy) can decrease the respiratory rate and increase the tidal volume by 150% (deep breathing pattern) [60–64]. Nevertheless, a higher concentration can lead to dizziness and sleepiness [65]. Another option would be the nebulization of a drug formulation in heliox. This low-density gas mixture has the ability to reduce flow resistance, in consequence allowing more aerosol to be deposited in the alveoli region (Figure 2) [66]. The CO_2_ has been co-administered in nebulized inhaled chemotherapy trials and inhaled gene therapy trials.

## 4. Aerosol Drug Formulation

Aerosol therapies have been tested and have already been used for decades [67]. It has been established that for the nanoparticles to be efficiently deposited and absorbed through the airways, they have to begin their journey from the upper airways on a scale of no more than 3.5 μm. This is necessary as due to the airway humidity; they will expand, and when they reach the alveoli, they have to be small enough to be deposited [68]. Larger particles (>5 μm) tend to deposit to the upper airways and not deposit to the alveoli. The macrophages have the ability to phagocytize particles 6 times their size, but the nanoparticles have to be equal in size in all axes. In the case were an axis is larger in size and the particle is not spherical, the macrophages will not be able to take up the nanoparticle, as usually happens with several types of fibers [69]. Another factor affecting the uptake from the site of deposition is the local transporters. This is a field under investigation, since different nano-molecules interact differently when they come in contact with local transporters. In addition to this observation, it should be mentioned that there are different transporters in different sites of the airways. When designing such an aerosol formulation, we have to be aware of the local transporters [48]. Moreover, another field of investigation is gene expression regulation. In the study by Leclerc *et al.* [47], more than 380 genes of the airway epithelium and lung cancer were investigated. It was observed that a number of genes are activated in cancer cells and others in the normal airway epithelium, indicating that aerosol therapy can become even more targeted. At this point it should be stated that aerosol targeted therapy has already been investigated in the form of adding ligands to the inhaled nanoparticles. A new form of nanoparticles has been created—the so-called “nanocomplexes”. Instead of having a cytotoxic agent administered alone or encapsulated (liposome, polyethylene glycol-PEG coated), an addition of a ligand further targeted the nanocomplexes to bind with specific receptors of cancer cells [28,70–72]. Further investigation of the nanocomplexes created a “stealth” drug formulation. This so-called “stealth” molecule has the ability to bypass the defense mechanisms of the airways, thus providing sustain release to a drug formulation [73]. Regarding aerosol gene therapy, the cationic polymers established effectiveness, based on their efficient binding to airway epithelial cells, as previously stated [34,37,43]. In addition, they do not form neutralizing antibodies, making them ideal for repeated administration. However, safety issues are to be presented in the following sections with reference, when necessary, to viral vector systems.

### 4.1. Aerosolized Vectors Safety Concerns

**Figure 3:**
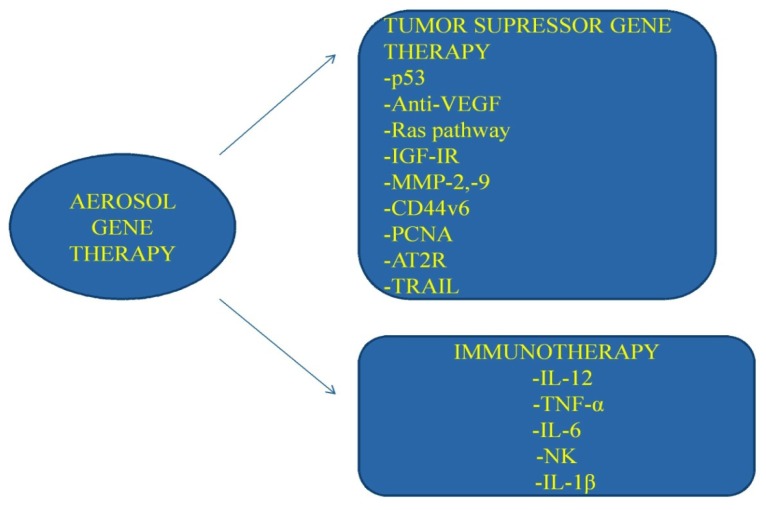
Gene therapy aerosol modalities. Figure by Paul Zarogoulidis.

In this section, all previous delivered nanocomplexes are presented with an emphasis on safety. Inhaled therapy safety concerns can be divided in two parts. The first consists of the drug formulation interaction with the medical personal administering the aerosol compound, and the second part, the drug formulation interaction with the patients or animal. Previous studies indicated that chemotherapeutic nanocomplexes induce bronchospasm, edema, hemoptysis and fibrotic lesions to the lungs [30,31] (Table 1, Figure 3).

Therefore, protection measures were necessary for the medical personnel. The most efficient protection measure was the administration of the inhaled compound in a high-efficiency particulate air tent with filter (HEPA). The drug compound administration efficiency was measured by Wittgen *et al.* [27], when the filter from a HEPA was used to investigate the drug compound (platinum analog) that was not inhaled from the patients. In this way, it was observed that only minimum non-toxic concentrations were diffused in the patients’ surrounding environment. Although the system used for aerosol administration in this study delivered the aerosol drug formulation efficiently, nevertheless, it provided us with the proper efficient measures that should be used to administer this treatment modality. Regarding the animal experiments, in almost all of the studies, a plastic cage was used to administer the aerosol compound, and therefore the medical personnel were safe [41,74]. Methods of establishing whether a compound affected the respiratory capacity of the patients were the: respiratory function measurement with forced expiratory capacity in 1 s (FEV1); forced vital capacity (FVC); diffusing capacity of the lung for carbon monoxide (DLCO), and the six minute walking test (6MWT). There were studies where these measurements took place before and after the aerosol administration [18,29]. In some studies, in order to protect the patients from possible adverse effects, inhaled bronchodilators and corticosteroids were administered before the aerosol [18,29,31]. The most observed adverse effects were intermittent fever and cough that fell after a maximum of 3 days. The intensity and duration was less for the group of patients that had pretreatment. Regarding the animals’ safety evaluation, the weight, bronchoalveolar lavage fluid (BALF) and immunohistological examinations were mostly used. There are no clinical data regarding the toxicity that is expected with aerosol gene therapy in humans. There are indications with several animal experiments; however, the human respiratory anatomy and defense mechanisms are different. The toxicity will derive only from the vector delivery system and, in specific, the non-viral vector system. Nevertheless, there are encouraging results from several studies that a non-viral biodegradable system could release efficiently the plasmid to the nucleus of the cells without inducing inflammation to the normal tissue. In addition, at this section it should be stated that only large molecule drug formulations have been accused of inducing bronchoconstriction, regardless of the toxicity of a molecule [75]. In order to have proper follow-up of inhaled gene therapy in lung cancer patients, lung function tests (FEV1, FVC, DLCO, 6MWT) and BALF has to be performed before and after aerosol gene therapy administration. BALF has to be evaluated at least one time.

#### 4.1.1. PEI

Polyethyleneimine (PEI) belongs to the cationic polymer family. They are constructed either as linear or branched and they have been used with low (2 kDa) and high (25 kDa) molecular weight. PEI can efficiently attach to the airway epithelial cells and efficiently introduce DNA in the cell nucleus [76]. PEI protects the DNA against DNAse degradation and protects the non-viral-DNA nanocomplexes from the sheer forces of nebulization [77,78]. Furthermore, PEI can efficiently deliver the DNA to tumor cells, and resist to surfactant inhibition [79]. In the study by Gautam *et al.* [37], large molecule branched PEI (PEI/CAT N:P ratio 15:1, 25 kDa) were used as a non-viral vector for efficient DNA delivery. Histological analysis and myeloperoxidase assay were performed to investigate inflammation. Histological analysis did not reveal inflammation and abnormal fibrotic tissue formation. Myeloperoxidase assay also did not reveal elevated values in azurophilic granules. This aerosol gene nanocomplex demonstrated safety. In the study by Zamora-Avila *et al.* [38], large molecule branched PEI (PEI/RNAi N:P ratio 10:1, 25 kDa) were administered and histological analysis was performed. No acute inflammation was observed. In another study by Gautam *et al.* [80], again large molecule branched PEI (PEI/p53 N:P ratio 10:1 25 kDa) were administered and no acute inflammation was observed; however safety was not properly investigated as it was not the endpoint of the study. In another study by Gautam *et al.* [43], large molecular weight branched PEI (PEI/p53 N:P ratio 10:1, 2 kDa) were investigated. Adverse histological findings regarding the PEI toxicity are stated, however, this was not the study endpoint. In addition, in two studies by Gautam *et al.* [63,64], the methodology of PEI construction, safety evaluation, gene transfection efficiency and nanocomplexes administration is thoroughly presented. In the study by Koshkina *et al.* [81], large molecular weight branched PEI (PEI/p53 N:P ratio 10:1, 25 kDa) were investigated as aerosol and intravenous gene therapy administration. The purpose of the study was to elicit safety and efficiency differences. In the aerosol treatment modality, as expected, the sequence of the organ formulation distribution and transgene expression was as follows: lungs > heart > blood > spleen > liver > kidney. Whereas, in the intravenous administration, the order was liver > spleen > blood > lung > heart > kidney. Once again, this sequence deposition demonstrates proof of concept for efficiency of local aerosol treatment modality. No acute inflammation was reported; nevertheless, this parameter was not investigated in the study, since the constructed nanocomplex had been previously investigated for safety [37,63,64]. In another study by Densmore *et al.* [61], large molecular weight branched PEI (PEI/p53 or p53CD (1-366) N:P ratio 10:1, 25 kDa) were investigated. Histological analysis for aerosol PEI toxicity revealed no acute inflammatory responses. However, it should be mentioned that mice receiving PEI aerosol presented a higher reduction in weight in comparison to untreated. In the study by Hasenpusch *et al.* [82], large molecular weight PEI (PEI/BC-819, 25 kDa) were investigated. No acute inflammatory responses were reported, although safety was not the prime endpoint. In the study by Duan *et al.* [83], the large molecular weight PEI (PEI:IL-12, 25 kDa) were investigated to deliver pCAGG plasmid containing murine IL-12 as aerosol gene therapy with or without chemotherapeutic agents. Although the systemic administration of IL-12 results in systemic toxicity, no acute inflammation was presented, as the nanocomplex achieved efficient concentrations on the tumor site. It has been previously demonstrated that bacterial plasmids due to the unmethylated CpG motifs induce cytokine production and activate B-cell response and natural killer (NK) activity [84]. Cationic lipids are known to induce mild cytokine production [85]; however the cationic-lipid/DNA nanocomplex tends to induce increased cytokine production [85,86]. Safety of PEI was investigated by Gautam *et al.* [34], by measuring immune response with the administration of large molecule branched PEI(25 kDa). The tumor necrosis factor-α (TNF-α) and interleukin-1β (IL-1β) were measured in serum and bronchoalveolar lavage fluid (BALF). Indeed, TNF-α and IL-1β were found elevated in serum at 2 h after intravenous administration. These two cytokine markers were observed increased in BALF after 5–8 h, but nevertheless, in much lower values than in serum. In the study by Densmore *et al.* [78], large molecular weight PEI (PEI/pCMV-hGH, N:P ratio 10:1, 25 kDa) were administered by instillation and aerosol. In addition, the liposome-DNA (pCMV-hGH) was administered as instillation and aerosol. The pCMV-hGH expresses human growth hormone, and the purpose of the study was to evaluate the efficiency of gene transfection and vector stability. The ratio chosen for repeatable administration was 10:1, based on the low toxicity observed at this concentration. The results indicated similar efficiency (cationic lipids, cationic polymers) with low toxicity. In the study by Jia *et al.* [87,88], the nanocomplex PEI/pCAGGmIL-12 (N:P ratio 10:1) was created. No acute inflammatory response was observed as it has been previously presented with intravenous administration [89]. In this study, aerosol gene therapy as immunotherapy was investigated and the administered nanocomplex of PEI/pCAGGmIL-12 demonstrated safety.

#### 4.1.2. cPEI

The method of ultrafiltration was used by Davies *et al.* [62] to create the nanocomplex of pCIKLux/PEI (pDNA/PEI N:P ratio 10:1 cPEI). Investigation of safety was performed with bronchoalveolar lavage fluid and histological analysis. In addition, a comparison of safety was performed between instillation and aerosol administration of aerosol gene therapy. No acute inflammation was observed for the group of aerosol administration. Only mild hemorrhagic edema between alveolar spaces and mild vascular congestion locally in some capillaries was observed. In contrast, the instillation method presented interstitial foci and necrosis due to neutrophil and lymphocyte rally. The extent of the damage was dose related. These results were attributed to the methodological disadvantage, where a large concentration of the non-viral vector comes in contact in one specific site and does not diffuse properly to the whole lung parenchyma. However, the new nanocomplex was more efficient and safe as aerosol gene therapy with a lower N:P ratio than the previous studies (10:1).

#### 4.1.3. GPEI

Further exploration of PEI resulted in the creation of large molecular weight glucosylated PEI (25 kDa) by Kim *et al.* [41]. The concept was to increase hydrophilicity of PEI and thus decrease the toxicity. It has been presented that the PEI toxicity is due to the prime amino group that occupies more than 30% of the molecule structure. Low toxicity was presented. In the study by Minai-Tehrani *et al.* [74], previously created GPEI non-viral vectors were administered as GPEI/Akt1 nanocomplex. A correlation of the results was made with naphthalene-induced inflammation in Clara cells. The GPEI presented safety in comparison to naphthalene with histological analysis. In another study by Tehrani *et al.* [90], large molecular weight glucosylated PEI (PEI/Akt WT or KD, 25 kDa) were investigated. No acute inflammatory toxicity was reported as already stated in the study by Kim *et al.* [41]. In the study by Hwang *et al.* [91], large molecular weight glucosylated PEI (PEI/PDCD4, 25 kDa) was prepared and investigated as in previous reported studies [41,90]. No acute inflammation and cytokine response was observed as stated in previous similar studies.

#### 4.1.4. PEI/PEG Noncomplex

Another approach to nanocomplex coating was the addition of poly (ethylene glycol) (PEG) by Jere *et al.* [92]. A new nanocomplex of low molecular weight PEI and poly (ethylene glycol) (PEG) (PAE/Akt1) was prepared and investigated. The newly formed nanocomplex was compared for safety with the already used non-viral vector PEI/Akt1 (high molecular weight 25 kDa), PAE presented less toxicity in comparison to PEI with cell proliferation assay and trypan blue dye exclusion. Another group by Xu *et al.* [93] again investigated a novel vector from PEI with PEG coating and Akt1 siRNA. The vector was degradable poly (ester amine) copolymer. Safety of the nanocomplex was investigated with BALF, lactate dehydrogenase (LDH) and histological assay. No acute inflammatory response was reported, probably due to the degradable property of the non-viral vector, as previously reported [94].

#### 4.1.5. Tetrafunctional Amphiphilic Block Copolymer 704

A new synthetic non-viral vector combines the properties of cationic vectors and those of non-ionic amphiphilic polymers. It consists of four polyethylene oxide/polypropylene oxide blocks centered on an ethylene diamine moiety. Efficient gene transfection was previously established in skeletal and heart muscles [95]. 704 synthetic vector was conjugated with fraktaline (CX3CL1), a powerful chemokine, the concept was to recruit and activate strong anti-tumor immune response. The nanocomplex was investigated by instillation administration and aerosol through a microsprayer^®^ (Penn Century) [96]. Histochemistry, histological assay and IL-6 measurements were used to evaluate inflammation in both administration modalities. Il-6 levels were higher for the instillation method. No acute inflammation was observed; nevertheless, increased perivascular and peribronchial infiltration were observed in the instillation modality in comparison to aerosol.

#### 4.1.6. Adenoviral Vector Encoding Murine GM-CSF

In the study by Xing *et al.* [97], human type 5 adenovirus genome with plasmid PACCMVmGM-CSF was instilled in mice. BALF, cytological examination and histopathology assay were performed. The group receiving only adenoviral vector presented mild neutrophilic and lymphocytic accumulation in BALF within the first 4 days, as in a previous study [98]. In addition, low levels of TNF-α were detected in some BALF samples, but decreased over a period of 4 days. Moreover, when AD5-PACCMVmGM-CSF was administered, in short time, low levels of TNF-α were detected, but in addition, a cascade of inflammation response was stimulated. Eosinophilia, neutrophilia, increased macrophage accumulation peribronchially and perivascularly were observed. Finally, after 12 days of the nanocomplex administration, severe fibrotic reactions led to shrinkage and destruction of the lungs. In this study, it is presented that the instillation of the adenovirus vector is safe, and the severe cytokine stimulation was due to the administration of the plasmid PACCMVmGM-CSF. Previously in the study by Arndt *et al.* [99], inhaled GM-CSF was administered as aerosol in humans and severe adverse effects occurred with reduction in the respiratory capacity of the patients, and destruction of the normal lung architecture and diffuse bilateral infiltration formation.

#### 4.1.7. dTAT

Through dimerization of HIV1-TAT, Kawabata *et al.* [71] formulated the dTAT vector. The vectors dTAT and PEI (large molecular weight branched, 25 kDa) were conjugated to a plasmid encoding Angiotensin-2-Receptor (pAT2R) and TNF-related apoptosis inducing ligand (pTRAIL). AT2R deficiency is associated with tumorigenesis in lung and colon [100,101], TRAIL is activating an anticancer pathway by inducing cell apoptosis [102,103]. The nanocomplexes were administered as aerosol by intratracheal spraying. Lower toxicity was observed for the dTAT vector in comparison with PEI. The dTAT also demonstrated transgene efficiency and additional glucose to the nanocomplex (dTAT/AT2R or TRAIL) caused further augmentation of this transfection. The dTAT vector is a safe vector for aerosol gene therapy; no dose escalation toxicity has been observed [71].

#### 4.1.8. Liposome-pDNA

In the study by Manunta *et al.* [36], the liposome vector (DHDTMA/DOPE) and pEGFP-N1 (enhanced green fluorescent protein) was nebulized. The nanocomplexes produced were positively charged and no acute inflammation response was reported. The cell viability results demonstrate data that this vector can be used for efficient gene transfection in cystic fibrosis and other respiratory applications.

#### 4.1.9. UAC

In the study by Jin *et al.* [104], imidazole ring-containing urocanic acid-modified chitosan (UAC, 100 kDa) was investigated as a vector. Chitosan has proven a safe gene carrier, due to the biocompatibility and biodegradability properties, and it demonstrates efficient gene transfection [105]. The UAC/programmed cell death protein-4 (PDCD4) nanocomplex was created and investigated for safety. Both chitosan/DNA and UAC/DNA were administered as aerosol, with a cell viability of >90% for both vectors, however the UAC/DNA nanocomplex demonstrated higher transgene efficiency, probably due to the proton sponge activity. Chitosan does not demonstrate immunogenicity and has excellent biocompatibility [106]. However, the large molecular weight, low solubility and low gene transfection in comparison to PEI brought them in the second line of vector systems, until the UAC was created. In the study by Okamoto *et al.* [107], the nanocomplex of chitosan/pDNA (interferon-β) was investigated as dry powder and instillation. This study is a critical study since it presents the proof of concept for preparation of a nanocomplex as dry powder. The chitosan cationic vector had low-molecular-weight (Mw = 2000–5000) [108], compared to another attempt [104]. The shape of the powder fiber was rectangular regardless of pDNA type, and the nanocomplex was taken up by endocytosis. The dry powder formulation is safe; however, instillation probably to the high local concentration accumulation of chitosan was not effective, in the sense of not properly diffusing the formulation to a large alveoli area. No acute inflammation was presented. The dry powder nanocomplex presented favorable results with less concentration. However, in the study by Huang *et al.* [109], additional investigation of chitosan nanoparticles for aerosol delivery was performed. Safety was assessed with bronchoalveolar lavage fluid-protein (BALF-P), lactate dehydrogenase activity (LDH) (BALF-LDH), myeloperoxidase activity (MPO), leukocyte migration and histopathological examination. The inflammatory markers were increased after 4 mg/kg intratracheal administration as aerosol (chitosan), compared to the control group (air inhalation). Inflammation observed was in the form of leukocyte infiltration, polymorphonuclear cell (neutrophils) and macrophage accumulation in lung tissue. Nevertheless, the inflammation activity was less compared to the bacterial liposaccharide (LPS) treatment. The LPS caused higher inflammatory stimulation for 4 mg/kg administration for the same amount of chitosan. The chitosan nanoparticle has a strong positive surface charge (+45 mV) and density of 0.38 g/cm^3^. The cationic polyelectrolyte property, with the high degree of deacetylation is responsible for the mild inflammatory activity presented *in vitro*. In another study by Takeuchi *et al.* [110], submicron-sized liposome (ssLip) were conjugated to chitosan molecules and formulated the ssCS-Lip nanocomplex. No adverse effects were observed with the administration. Moreover, effective mucosa penetration was observed, making this nanocomplex an excellent candidate for aerosol administration gene therapy. The formulation reached the bloodstream, although the exact mechanism of the drug release is not yet fully understood.

#### 4.1.10. AND

In the study by Zou *et al.* [42], protamine sulfate, L-polylysine, and polyethyleneimine were prepared as the vector called “AND”. The nanocomplex AND/p53 was created and administered intratracheally as aerosol. The vector was administered alone or as AND/p53 nanocomplex and dose lethal toxicity was investigated. The aim of the study was to investigate the alternative of combining two to three cationic polymers and establish the higher gene transfection nanocomplex. The best combination was found to be polylysine and protamine at weight ratio of 8:1 [42]. Toxicity was dose dependent, a concentration of 22.4 mg/m^2^ demonstrated average toxicity ≤3. Inflammatory infiltrates with lymphocytes, pneumocytes and histiocytes were observed, but resolved after 14 days. Finally, when the dose was increased to 112 mg/m^2^, the toxicity reached grade 7, the lung architecture was lost and the scar tissue damage were irreversible. At low dosages <22.4 mg/m^2^ the AND presented safety and efficient gene transfection.

#### 4.1.11. Lentiviral shOPN

Lentiviruses have been also used as vectors, by Yu *et al.* [21], where a lentivirus was conjugated with shRNA, the small hairpin osteopontin (shOPN). The nanocomplex was administered as an aerosol. The viral vector was evaluated alone and as a nanocomplex Lentivirus/shOPN. The lentiviruses are known to present high gene transfection, both *in vitro* and *in vivo* [111]. No acute inflammatory response was reported and therefore the nanocomplex is considered safe. Another possible explanation could be the multiple pathways that are down-regulated with the specific nanocomplex (vascular endothelial growth factor (VEGF), expression of proliferating cell nuclear antigen (PCNA), CD44 variant 6 (CD44v6), and matrix metalloproteinase-2,-9(MMP-2,-9)).

### 4.2. Future Nanocomplexes

In this section, nanocomplexes with a possible application as vector or drug releasing system for aerosol gene therapy are presented (Figure 2).

#### 4.2.1. Cross-Linked Small PEIs

Another approach was investigated by Thomas *et al.* [112], to create a vector with less systemic side effects, and therefore small PEIs 2 kDa with biodegradable linkages were tested *in vitro* and *in vivo.* Biodegradable linkages consist of the following: (a) esters, (b) amides, (c) orthoesters, (d) acetals, (e) glycosides and (f) bisulfides [113]. The concept was to produce a nanocomplex that would biodegrade in a short time and therefore the interaction between the cells will induce inflammatory stimulation. The cross-linked PEIs demonstrated 95% cell viability [112]. The efficiency in *in vitro* was presented up to 550- and in *in vivo* up to 80-, without any toxicity. It is known that small PEIs are not efficient (2 kDa). On the other hand the large PEIs (25 kDa) are toxic, therefore the cross-linked biodegradable 2 kDa PEIs demonstrated that short time local administration was safe and efficient (gene transfection) without any toxic adverse effects. In another study by Wang *et al.* [114], small molecular weight PEI (2 kDa) were synthesized and structured with biscarbamate linkages, the PEI-ET nanocomplex was constructed (Mn: 1220, Mw: 2895). The non-viral vector presented lower toxicity in comparison to PEI 25 kDa and efficient gene expression in three different cell lines. The optimal ratio *w*/*w* for efficient gene transfection (3.4-higher than 25 kDa PEI) was 20. Nevertheless, concentration-dependent toxicity was observed at >50 μg/mL. This novel nanocomplex still remains to be tested for aerosol stability.

#### 4.2.2. PEIs with PEG Shielding

In the study by Uchida *et al.* [115], PEG was added to PEIs as a shield to stabilize the nonspecific toxic interactions of the PEI. The polyplexes have a high surface positive charge, which is known to interact with the airway epithelial cells. Nevertheless, the addition of PEG with the neutralizing and hydrophilic properties decreases the transgene expression [116]. Therefore, an attempt was made to increase the N/P ratio. The result was higher transgene efficiency, but with additional toxicity. The authors created the nanocomplex PEG-block-PAsp (DET) and homo PAsp (DET). The structure had high efficiency with minimal toxicity. The safety was evaluated with several markers, such as: (a) IL-6, (b) cyclooxygenase, (c) IL-10, (d) Tumor necrosis factor (TNF), and (e) C-reactive protein (CRP). No acute inflammation was observed with additional, immunohistochemistry assays, in several organs. The possible explanation is again the biodegradable property of the PAsp (DET). Further investigation with microsprayer^®^ intratracheal administration of PEG-block-PAsp (DET) and homo PAsp (DET) 0/100 to the airways of animals presented acute inflammation (pro-inflammatory activation and increased cycloxigenase-2 expression). The optimal safety balance between the PEG/PEI (efficiency/toxicity) ratio is 50/50. In the study by Fan *et al.* [117], another form of PEI shielding was investigated by adding the nonionic amphiphilic surfactant polyether-Pluronic^®^. This nanocomplex consists of hydrophilic ethylene oxide and hydrophobic propylene oxide blocks [117]. The addition of Pluronics^®^ demonstrated enhanced DNA cellular uptake, nuclear translocation, and gene expression with low toxicity. The efficiency was directly associated with the Pluronics^®^ concentration. This nanocomplex structure remains to be tested as aerosol. In the study by Jiang *et al.* [118], another form of active transport was investigated by targeting the mannose receptor of macrophages, and the nanocomplex structure of mannan-PEG-l-α-phosphatidylethanolamine (PE) was created. Efficient transgene expression was observed, making this structure ideal for macrophage targeting and therefore provided proof of concept for macrophages being the carrier of a gene treatment through lymphatic circulation [119]. Cell viability assay confirmed 100% safety; nevertheless, this structure also remains to be tested as aerosol. In the study by Zeng *et al.* [120], the concept of creating shield for an adenovirus vector was investigated. A cationic PEG derivative (APC) was formulated to coat the adenovirus 5 and presented effective protection against neutralizing antibodies (Nabs). The structure of Ad5/APC-PEG also presented high transgene expression. PEIs with low molecular weight 2 kDa and high molecular weight 5 kDa were compared with the APC coating for safety in multiple cell lines. The APC presented low toxicity comparable to PEI 2 kDa, and even lower to 25 kDa PEIs. This novel formulation remains to be tested as aerosol.

#### 4.2.3. Solvoplex

A future methodology was proposed for gene therapy, by Schughart *et al.* [121,122]. The following solvoplexes presented high transgene expression: (a) di-*n*-propylsulfoxide (DPSO), (b) dimethylsulfoxide (DMSO), (c) tetramethylurea (TMU), and butylmethylsulfoxide (BMSO). The DPSO/DNA nanocomplex presented high transgene expression and stability either administered intratracheally directly in the airways or as aerosol with a microsprayer^®^ [121]. The microsprayer^®^ prevents the degradation of the solvoplex/DNA complex, in contrast to jet nebulization. Repeated administration of solvoplexes is possible with low toxicity. The safety evaluation was performed with histopathology assay, alanine transaminase and asparagine transaminase. Administration of DPSO-solvolex at concentrations of 5%, 10% and 15% demonstrated that moderate inflammation occurred at 15% with edema and focal lesions which were resolved within 7 days.

#### 4.2.4. APTES

In the study by Cheang *et al.* [123], a new type of silicon nanoparticle the aminopropyltriethoxysilane-functionalized silicon dioxide nanoparticle was developed (APTES-SiNPs). This nanocomplex has previously demonstrated safety and efficiency, since they present low toxicity, excretion via the renal route, and they are biodegradable [124,125]. The quaternized APTES derivatives present less toxicity than regular APTES, because they have the property of maintaining the cationic charge inside the polymer covered by hydroxyl groups on the surface. In the study by Cheang *et al.* [123], the APTES nanoparticles were compared with the Lipofectamine^®^ 2000 and the results did not present any toxicity for the quaternized APTES nanocomplex in human cell lines, compared to Lipofectamine^®^ 2000. The APTES nanocomplex maintains the low level of toxicity at high concentrations and prolonged local deposition. However, the APTES has been tested only *in vitro* and therefore remains to be tested *in vivo* and as an aerosol delivery system.

#### 4.2.5. PLGA Delivery System for Immunotherapy

In the study by Ma *et al.* [126], the poly(dl-lactide-co-glycolide) (PLGA) nanoparticle delivery system was developed to encapsulate tumor antigenic peptides [126]. The nanocomplex has immunomodulatory properties of activating and stimulating the T-lymphocytes against tumor cells. Higher efficiency was demonstrated *in vivo* for PLGA–NPs delivery systems. Further investigation led to the efficient encapsulation of three peptides to the PLGA-NPs and loaded to the dendritic cells (DCs). A powerful response of cytotoxic T lymphocytes (CTLs) was observed. The nanocomplex was evaluated with cell viability assay for safety, and remains to be tested *in vivo.* In the study by Li *et al.* [127], a PLGA nanocomplex was conjugated with PEG, the endpoint of the authors was the investigation of the nanoparticle properties and bio-distribution. Further investigation has to be performed for PLGA nanocomplexes for stability as aerosol administration.

#### 4.2.6. Gene and Chemotherapy in One Complex (mPEG-PCL-g-PEI)

In the study by Shi *et al.* [128], the polyethylene glycol-poly ɛ-caprolactone-polyethylamine (mPEG-PCL-g-PEI) an amphiphilic triblock copolymer was synthesized to deliver simultaneously doxorubicin and plasmidDNA. The complex is biodegradable and it was tested *in vitro* in several concentrations of mPEG-PCL-g-PEI (2000-2000-2000, 2000-6000-2000 and 5000-2000-2000). The mPEG-PCL-g-PEI copolymer 5000-2000-2000 has the highest transgene efficiency, while the formulation 2000-2000-2000 had the highest drug-loading capability. Cytotoxicity was observed in higher concentrations, but it differed between tested cell lines. The nanocomplex remains to be tested *in vivo*. The concept of gene therapy and chemotherapy co-administration could bring a new era to multimodality lung cancer treatment.

#### 4.2.7. Carbonate Apatite nano-Carriers

Several techniques were investigated in order to develop the nanocomplex of an optimal size and efficiently induce gene transfection by Chowdhury *et al.* [129]. The basic parameters: Ph of buffered solution and incubation temperature, were investigated and presented the optimal values to develop a highly efficient nanoparticle gene delivery system with a possible application for aerosol gene therapy delivery. Carbonate apatites are biodegradable nanoparticles that have presented efficient transgene expression [129,130]. Almost no toxicity has been observed *in vitro* when the complex of carbon apatite—siRNA—was delivered to cell cultures [130]. In another study by Hossain *et al.* [131], ph-sensitive carbon apatite nanocomplex was developed. There was no acute inflammation reported (cell viability assay), and the nanocomplex had the property of a ph-drug release system. When the nanocomplex contacts pH < 7, it “activates” and delivers the RNA load. This drug delivery system again is an excellent candidate for aerosol gene therapy administration, as it is activated with the acidic cancer cells pH ≤ 6.5. This application could be incorporated as aerosol gene therapy for efficiently in endobronchial tumors.

#### 4.2.8. F-AL-Ad5

In the study by Zhong *et al.* [72], a nanocomplex conjugating an adenovirus vector and anionic liposome was investigated. The F-AL-Ad complex was investigated in normal airway epithelial cells and not in cancer cells, so the efficiency of the treatment still remains to be tested on a cancer model. It is known that folate receptors are over-expressed in a variety of tumors and therefore this nanocomplex could be used in aerosol gene therapy with the addition of a plasmid [132]. The nanocomplex of adenovirus vector-5 and anionic liposome has been previously created, and in the present study, folate was added to the nanostructure, to create F-AL-Ad complex. The complex was not efficient when administered basolaterally, because the folate receptors are absent at the basolateral side, and therefore affected only the cancer cells. No acute toxicity was reported and this nanocomplex is an excellent candidate for receptor targeting vector. It remains to be tested as aerosol.

#### 4.2.9. Amino Acids to Enhance the Aerosol Deposition

Amino acids have been proposed to enhance the efficiency of a delivery system through nebulization. Therefore, the amino acids arginine, aspartic acid, threonine and phenylalanine were investigated as to whether they could enhance the stability of aerosol nanocomplexes by Li *et al.* [133]. Indeed, the arginine, aspartic acid and threonine addition produced more uniform particles, in specific arginine and phenylalanine demonstrated optimal powder aerolization. The addition of these two amino acids can influence the structural integrity of the dry powder formulation and therefore merit being incorporated in the nanocomplex structure; however, they decrease the gene transfection. Therefore, a balance has to be investigated for the optimal concentration within a vector/DNA nanocomplex. In the study of Li *et al.* [134], another amino acid, leucine, was investigated and further enhanced the aerosol dispersion and deposition. However, again, the amino acid leucine negatively influenced the biological activity of the gene vector. The addition of the reported amino acids did not present any inflammatory activity.

#### 4.2.10. GOLD Nanoparticles

In the study by Puvanakrishnan *et al.* [135], gold nanoparticles (GNPs) were investigated as pegylated gold nanoshells (GNSs) and gold nanorods (GNRs) and demonstrated a safe profile. These nanovectors presented low toxicity locally on several organs that were dose-dependent when injected systematically. No necrosis was observed. The GNPs are non-toxic, stable and pose unique optical and thermal properties [136]; in addition, they are PEG coated and therefore have the “stealth” ability to bypass several defense mechanisms [137], making them an ideal nanocomplex to bypass the respiratory defense mechanisms. This nanovector could therefore be utilized as an aerosol gene delivery system; however, multiple experiments should be performed *in vitro* in airway epithelial cells and *in vivo* animal studies. The possible aerosol nanocomplex has to be tested for stability within the sheer forces of nebulization. Moreover, gold nanoparticle sensors have recently been used efficiently for classification of lung cancer [3,6].

#### 4.2.11. PH-Release System

In the study by Li *et al.* [138], a ph-sensitive delivery system was investigated. The anionic DNA, cationic liposomes, and o-carboxymethyl-chitosan (CMCS)-cationic liposome-coated DNA/protamine/ DNA complexes (CLDPD) were created. The nanocomplex is designed to deliver the gene load, based on the ph of the cells with which it comes into contact. In specific, the nanocomplex is activated not in the blood serum with a pH 7.4, but only when the formulation came in contact with the tumor cells with a pH of 6.5. Since the drug formulation will only be activated when it comes in contact with the tumor cells, therefore it does not interact with the normal cells. The CMCS-CLDPD also demonstrated less cytotoxicity compared to PEI/DNA (N:P ratio 10:1), due to the biodegradable property [138]. However, this method still remains to be tested for aerosol stability. No acute inflammation was observed and cell viability was increased as observed to other chitosan derivatives [107].

#### 4.2.12. Lactoferin Nanoparticles

Lactoferin (Lf) is an iron binding glycoprotein that resembles transferrins (Tf) [139]. It is found in several fluids of the body [140]. Lf was previously conjugated with DNA and formed a nanocomplex able to efficiently deliver gene therapy. The Lf/DNA nanocomplex demonstrated even higher efficiency than Tf/Liposome/DNA [141]. In another study by Gupta *et al.* [142], Lf nanoparticle was conjugated with ceruloplasmin and superparamagnetic iron oxide and increased cell surface binding was observed. The Lf can be used as a target-delivery nanocomplex. In addition, different cells, such as monocytes/macrophages, lymphocytes, platelets, osteoblasts, chrondrocytes and hepatocytes, express receptors efficient to Lf, again demonstrating proof of concept that macrophages through lymphatic circulation can enhance an anticancer treatment [143,144]. Efficiency and safety of this carrier is still to be evaluated as aerosol gene therapy.

#### 4.2.13. Mannosylated Liposomes

A novel formulation of mannosylated polyethylene glycol-phosphatidylethanolamine (M-PEG-PE) was developed and intravenously injected by Kong *et al.* [145]. No acute inflammation was observed and cell viability was observed. In addition, efficient gene transfection was established, and PEG coating protects can protect the nanocomplex from the defense mechanisms of the respiratory system, namely macrophages. This nanocomplex could therefore be evaluated as aerosol gene therapy; nevertheless, caution should be taken in the case where adverse effects should be presented from the respiratory system (bronchospasm).

## 5. Conclusions

Nanocomplexes have the ability due to their small size to diffuse through tight junctions and cell membranes, while other larger particles fail. It has been previously demonstrated that they deposit and accumulate at the tumor site for a longer time than uncoated drug formulations. The nanocomplexes can be used as the aerosol gene therapy treatment, with numerous multimodal approaches, most recently as early lung cancer detection system [2–6].

Vector systems (viral and non-viral) are used in gene therapy, each with specific properties, and therefore a different safety and gene transfection profile. The adenovirus vectors, although they have higher gene transfection, nevertheless tend to create neutralizing antibodies. Non-viral vectors also present higher affinity binding to the airway epithelial cells, in comparison to viral vectors [35,146]. In contrast, the non-viral vectors efficiently bind to the airway epithelial, but they do not present efficient transfection in comparison to the viral vectors. In addition, safety concerns have been raised concerning the non-viral vectors and in specific for PEI [147]. Another important aspect is the production of the two delivery systems, the viral vectors are produced with a high cost, in comparison to the low production cost of the non-viral vectors [148]. Moreover, an important aspect for inhaled gene therapy is the ability of the vector and nanocomplex to be delivered as either a dry powder or aerosol. The sheer forces of nebulization tend to degrade viral vectors. Cationic lipid vectors stabilize the DNA and therefore present high gene transfection; however, nebulization again degrades the nanocomplexes and reduces the degree of gene transfection. Nevertheless, many efforts towards modification of cationic lipids resulted in the development of lipid-based nanocomplexes with satisfactory gene transfection [149–151]. Adenoviruses (Ad), adeno-associated virus (AAV) have been used for aerosol gene therapy, with moderate gene transfection results. Regarding delivering a nanocomplex as aerosol gene therapy (with Ad and rAAV), the drug-formulations were observed to be efficiently diffused throughout the lung parenchyma [36,152]. In previous studies regarding inhaled chemotherapy, the safety was evaluated in patients and medical personnel [19].

No adverse effects were observed in the medical personnel; however, transient reduction in the respiratory function of patients was observed. Therefore preparation with bronchodilators and inhaled corticosteroids was administered, either pre or post the inhaled chemotherapy administration. High particulate air filters (HEPA) and other protection measures, such as plastic tents (patients) and cages for animals, were used as a protection from dispersed chemo agents to the environment. However, HEPA filter measurements did not reveal high chemotherapeutic agent dispersion. Regarding the inhaled gene therapy, although protection measures were followed in multiple studies, there are no robust data from human studies. The previously published studies efficiently constructed nanocomplex particles with the necessary properties for efficient aerosol delivery and deposition [68,69]. However, Ad and rAAV induce inflammation and stimulate cytokine production. Flu-like symptoms are also observed and therefore they should be administered with caution to an already compromised respiratory system. The non-viral vectors are therefore suitable for repeatable delivery. The viral vectors are not suitable for repeatable application and the most important issue is that they do not bind efficiently to the airway epithelial as the non-viral vectors. Aerosol delivery properties and systems have been previously analyzed for aerosol delivery in lung cancer [19]. Several modalities of aerosol distribution have been properly displayed by Beck *et al.* [152], and the microsprayer^®^(Penn Century) presented the most efficient aerosol distribution. Nevertheless, this method has not been tested in humans and it remains minimally invasive. Regarding nebulization, the Aeroneb^®^ nebulizer delivers aerosol efficiently with minimal nanocomplex degradation.

The cytotoxicity of the non-viral vectors (mainly polymers) is a consequence of the molecule aggregation on the cell surface, due to the strong electrostatic charge. This interaction vector-cell induces cell membrane instability. The optimal molecular weight for effective gene transfection is between 5 and 25 kDa; PEIs less than 1.8 kDa present almost no transfection. However, higher molecular weight of the PEI induces higher toxicity. Furthermore, the higher the N:P ratio the lower the size of PEI nanocomplexes [153]. Linear and branched PEI/DNA nanoparticle size depends on the method of construction (glucose/salt/solvent) [154]. Linear PEI present a more efficient gene transfection, but present higher toxicity compared to branched: branched presents higher effectiveness in delivering aerosol gene therapy [155].

There are delivery systems, RNA/DNA, currently under investigation the exosome mimetics. These extracellular vesicles (phospholipids) have been identified as carriers. They have the ability to affect many systems at once, therefore different components of the exosome mimetics (incorporation of multiple membrane proteins in liposomes) are to be investigated and explored in correlation with a target system [156]. Toxicity is also an issue due to the hydrophobicity. This vehicle has still remains to be tested for stability at nebulization and for *in vitro*–*in vivo* toxicity investigation.

The exosome mimetics have the ability to encapsulate different and multiple molecules and therefore target simultaneously several cells and pathways [156]. PLGA nanoparticles have been used as carriers to encapsulate tumor antigenic peptides with safety [126,157]. GOLD nanoparticles, which have PEG shielding, have unique thermal properties to be activated with thermal energy and presented cell viability [135]. Lf is another nanoparticle that can be used with safety as a non-viral vector for aerosol gene therapy [158]. Moreover, several efforts have been made towards creating a safe non-viral vector. Several nanocomplexes were investigated where small-molecule 2 kDa PEI were linked with biodegradable linkages, with additional PEG encapsulation [115–118].

Moreover, several efforts have been made to block or minimize the neutralizing antibodies created by viral vectors. These efforts succeeded only in reducing the numbers of neutralizing antibodies [159–161]. Another system currently under investigation is the “Sleeping Beauty”; actually an enzyme that recognizes the ends of a transposon. It utilizes the transposon as a vector, however, this system SB transposase/transposon could result in mutagenesis and further investigation is required [162].

Going through all the previous published data, there are sufficient data regarding safety of the non-viral vectors (PEI) and others are yet to be investigated *in vivo*. Proper safety evaluation requires that several parameters have to be assessed at the same time. The nebulizer, conditions of nebulization, amount of DNA and purity, specific reporter plasmid, vector concentration, nanocomplex coating, and methodology of construction. A non-viral system nanocomplex with biodegradable linkages and small-molecule PEG encapsulating nanocomplexes with or without additional surface ligands has to be pursued. A multifunctional nanocomplex is also welcomed, if it demonstrates safety and stability as an aerosol. Several methodologies for viral and non-viral delivery systems were previously presented; however, with current technology we should direct our efforts towards a multifunctional vector (sustained drug release, receptor target) and biodegradable non-viral vector.

## Figures and Tables

**Table 1:** Gene Therapy Studies.

Author	Gene	Evaluation	Toxicity	Protection	Inhalation Mode	Subjects	Reference
Hasenpusch *et al.* (2011)	PEI-BC-819	Histologic Bioluminescence	-	Inhalation Chamber	Nebulizer (spacer)	*In Vitro* *In Vivo*	[82]
Xu *et al.* (2008)	PEI + PEG Akt1 siRNA	BALF, LDH, IHC, Histologic, RTPCR, Western Blot	No toxicity	Nose only chamber	Aerosol	*In Vivo*	[93]
Jin *et al.* (2006)	UAC-PDCD4	Western Blot, IHC, TUNEL	Low toxicity	Nose only chamber	Patent Nebulizer No. 20304964	*In Vitro* *In Vivo*	[104]
Zou *et al.* (2012)	AND –p53sm	Weight, RT-PCR, extrusion precipitation	Dose dependent peribronchial inflammation	Accurately aerosol administration	Nebulizer	*In Vitro* *In Vivo*	[42]
Densmore *et al.* (2001)	PEI-p53/p53-CD(1-366)	Weight, histological, ELISA, IHC	No acute inflammatory response	Inhalation Chamber HEPA	Nebulizer + 5% CO_2_	*In Vitro*	[61]
Gautam *et al.* (2000)	PEI-p53	Histological, ELISA, weight	Low toxicity	Inhalation Chamber	Nebulizer + 5% CO_2_	*In Vitro* *In Vivo*	[37]
Okamoto *et al.* (2011)	Chitosan-Interferon-β	Scanning electron microscope, histological, weight	-	Intratracheal	Dry powder	*In vivo*	[107]
Tehrani *et al.* (2011)	GPEI-Akt1WT or KD	Western Blot, IHC, histopathological, CC10 marker	Low toxicity, correlated with naphthalene	Nose only chamber	Patent Nebulizer No. 20304964	*In Vivo*	[74]
Gautam *et al.* (2002)	PEI-p53	IHC, CAT IHV, vWF, VEGF-TSP-1 ELISA	-	Inhalation Chamber	Nebulizer + 5% CO_2_	*In Vitro* *In Vivo*	[42]
Tehrani *et al.* (2007)	GPEI-Akt1WT or KD	Western blot, IHC, Luciferase	Low toxicity	Nose only chamber	Patent Nebulizer No. 20304964	*In Vivo*	[90]
Kim *et al.*(2004)	GPEI-pcDNA3.0-PTEN	Western blot, IHC, Detection of Apoptosis, Immuno-precipitation and Kinase assays, TUNEL, GFP expression	Low toxicity	Nose only chamber	Patent Nebulizer No. 20304964	*In Vivo*	[41]
Koshkina *et al.* (2003)	PEI-p53	Southern Blot analysis, Andersen cascade impactor, RT-PCR, Genomic DNA isolation	Low toxicity	Inhalation Chamber	Nebulizer + 5% CO_2_	*In Vivo*	[81]
Hwang *et al.*(2007)	GPEI-PDCD4	Western Blot, IHC, TUNEL	Low toxicity	Nose only chamber	Patent Nebulizer No. 20304964	*In Vivo*	[91]
Frederiksen *et al.* (2000)	EGF-DNA complex	Receptor binding studies, Transfection experiments	-	-	-	*In Vitro*	[40]
Zamora-Avila *et al.* (2009)	PEI-RNA WT-1,2	RT-PCR, TUNEL, histology, weight	Low toxicity	Nose only chamber	Micro-mist Nebulizer	*In Vivo*	[38]
Jere *et al.* (2008)	PAE-shRNA (Akt1)	EFTEM, FACS, confocal Microscopy, Western Blot, RT-PCR	PAE Low toxicity *vs.* PEI	-	aerosol	*In Vitro* *In Vivo*	[92]
Densmore (2003)	Review	Review	Review	Review	Review	Review	[39]
Topical Gautam *et al.* (2003)	PEI-CAT	CAT, Luciferase, Histological, IHC, MPO, BALF	Toxicity concerns Presented for personnel and mice	Inhalation Chamber	Nebulizer + 5% CO_2_	*In Vivo*	[64]
Gautam *et al.* (2003)	PEI-p53CD(1-366)	IHC, ELISA, Tumor growth	-	Inhalation Chamber	Nebulizer + 5% CO_2_	*In Vivo*	[63]
Davies *et al.* (2008)	pCIKLux/PEI (cPEI)	BALF, Histological, Luciferase, GFP expression, Electron microscopy	Hunching, Pronounced piloerection, Weight loss 5-10%, Foci of interstitial inflammation, Hemorrhage, Necrosis	Inhalation Chamber	Nebulizer + 5% CO_2_/Instillation	*In Vivo*	[62]
Gautam *et al.* (2001)	PEI-DNA BGTC:DOPE-DNA DOTAP-Chol:DNA	TNF-a, IL-1β, MPO, PMN, Histology, Elisa, Weight, Luciferase, MPO, BALF	No Toxicity	Inhalation Chamber	Nebulizer + 5% CO_2_	*In Vivo*	[34]
Dong *et al.* (2007)	siRNA IGF-IR PEI	RT-PCR, Western Blot, Flow Cytometry, Cell Proliferation, Apoptotic Detection, TUNEL	-	Intratumoral	Intratumoral	*In Vivo*	[70]
Xing *et al.* (1996)	Human type 5 adenovirus with a CMV promoter	Northern hybridization analysis, RT-PCR, BALF,, Cytology, Histology, Elisa	Severe fibrotic reactions infiltrates of mono-nuclear cells, neutrophils and eosinophils	None	Instillation	*In Vivo*	[97]
Duan *et al.* (2005)	PEI:IL-12 ± IFX	Elisa, Fas/FasL, IHC, CD31, bFGF, PCNA, weight	-	-	Intranasal	*In Vivo*	[83]
Yu *et al.* (2010)	shOPN (recombinant lentivirus)	Western blot, IHC, Wound healing assay, VEGF, MMP-2, MMP-9, CD44v6, PCNA	-	Nose only chamber	Intranasal	*In Vivo*	[21]
Kawabata *et al.* (2012)	dTAT, PEI- AT2R, TRAIL	RT-PCR, TUNEL, Ki-67, IHC, Histology	PEI toxicity, but not for dTAT vector	-	Intratracheally	*In Vitro* *In Vivo*	[71]
Richard-Fiardo *et al.* (2011)	Amphiphilic Copolymer 704/Fraktalkine (CS3CL1)	IHC,CAT, IL-6, BALF, Histology, Western blot, IL-12, NK cells	No histological abnormalities, increased IL-6 after 6 hours, Mononuclear infiltration In perivascularly and Peribronchial zones	-	Instillation, Microsprayer	*In Vitro* *In Vivo*	[96]
Jia *et al.* (2002)	PEI:IL-12	Northern blot analysis, RT-PCR	Low toxicity	-	Intranasal instillation	*In Vitro* *In Vivo*	[88]
Jia *et al.* (2003)	PEI:IL-12	RT-PCR, IHC	Low toxicity	Plastic cage, HEPA	Nebulizer + 5% CO_2_	*In Vitro* *In Vivo*	[87]

MPO: myeloperoxidase; CAT: chroramphenicol acetyl transferase gene; PMN: polymorphonuclear leukocyte; BALF: bronchoalveolar lavage fluid; GFP: green fluorescent protein; TUNEL: Terminal deoxynucleotidyltransferase mediated dUTP Nick End Labeling assay; -: Not stated/presented; TNF-a: tumor necrosis factor-a; IL: interleukin; RT-PCR: real-time polymerase chain reaction; EFTEM: Energy-filtered transmission electron microscopy; CAT: Catalase; FACS: Flourescence activated cell sorting; BGTC:DOPE: guanidinium–cholesterol: dioleoylphosphatidyl–ethanolamine liposome; DOTAP-Chol: 1,2-dioleoyl-sn-glycero-3-trimethylammonium–propane-cholesterol; PEI: polyethylamine; GPEI: glucosylated polyethylamine; Akt1: protein kinase B; WT: wild type; KD: kinase- deficient; UAC: imidazole ring containing urocanic acid modified chitosan; AND: protamine sulfate, L-polylysine and polyethyleineimine; PEG: polyethylene glycol; CD31: antibody for vessel density; bFGF: basic fibroblast growth factor; PCNA: staining for tumor cell proliferation; Fas/FasL: type-II transmembrane protein that belongs to the tumor necrosis factor (TNF) family; OPN: osteopontin; PCNA: proliferating nuclear cell antigen; MMP-2,-9: Matrix Metalloproteinase.
