# Zarogouldis, P., *et al*., Vectors for Inhaled Gene Therapy in Lung Cancer. Application for Nano Oncology and Safety of Bio Nanotechnology. *Int. J. Mol. Sci.* 2012, *13*, 10828–10862

**DOI:** 10.3390/ijms131217290

**Published:** 2012-12-18

**Authors:** Paul Zarogoulidis, Nikos K. Karamanos, Konstantinos Porpodis, Kalliopi Domvri, Haidong Huang, Wolfgang Hohenforst-Schmidt, Eugene P. Goldberg, Konstantinos Zarogoulidis

**Affiliations:** 1 Pulmonary Department-Oncology Unit, “G. Papanikolaou” General Hospital, Aristotle University of Thessaloniki, Thessaloniki 57010, Greece; E-Mails: kporpodis@yahoo.gr (K.P.); kellybio4@hotmail.com (K.D.); zarog@med.auth.gr (K.Z.); 2 Pulmonary Department-Interventional Unit, “Ruhrland Klinik”, University of Essen, Essen 45239, Germany; 3 Laboratory of Biochemistry, University of Patras, Patras 25200, Greece; E-Mail: n.k.karamanos@upatras.gr; 4 Department of Respiratory diseases, Changhai hospital, Second Military Medical University, Shanghai 200433, China; E-Mail: hhdongbs@126.com; 5 II Medical Clinic, Hospital of Coburg, University of Wurzburg, Coburg 96450, Germany; E-Mail: w.h-s@gmx.de; 6 Biomaterials Science & Engineering, Department of Materials Science & Engineering, University of Florida, FL 32611, USA; E-Mail: egold@mse.ufl.edu

The authors wish to add this correction on their paper published in *IJMS*[1]. The first author’s name is misspelled and the correct name is Paul Zarogoulidis. In addition, the 6th author’s name is incorrect and should be corrected to Wolfgang Hohenforst-Schmidt. These errors have been amended in an amended version of the manuscript, which is available from the International Journal of Molecular Sciences website. The authors and publisher apologize for the inconvenience.

The corrected version can be accessed at: http://www.mdpi.com/1422-0067/13/12/17290/s1.
